# Spiritual Care Competence in Palliative Care: A Concept Analysis

**DOI:** 10.1007/s10943-025-02408-1

**Published:** 2025-08-08

**Authors:** Joana Coelho, Ana Querido, Cristina Costeira, Carlos Laranjeira

**Affiliations:** 1 School of Health Sciences, Polytechnic University of Leiria, Campus 2, Morro do Lena, Alto do Vieiro, Apartado 4137, 2411-901 Leiria, Portugal; 2 Centre for Innovative Care and Health Technology (ciTechCare), Polytechnic University of Leiria, Campus 5, Rua das Olhalvas, 2414-016 Leiria, Portugal; 3 RISE Health Research Group, University of Porto, 4200-450 Porto, Portugal; 4 Health Sciences Research Unit: Nursing (UICISA: E), Nursing School of Coimbra (ESEnfC), 3004-011 Coimbra, Portugal; 5 Comprehensive Health Research Centre (CHRC), University of Évora, 7000-801 Évora, Portugal

**Keywords:** Spirituality care competence, Concept analysis, End-of-life, Palliative care, Attributes

## Abstract

Care is a multidimensional concept that includes spirituality as a dynamic and integrative aspect of human experience. Integrating spirituality into clinical practice enables a more comprehensive response to the full spectrum of human needs, which is why spiritual care competence emerges as an aptitude that fits the profile and skills to be developed by those working on care provision. Regrettably, not enough attention has been paid to spiritual care competence largely due to the lack of knowledge of professionals and what this entails. Therefore, this study aims to define the main attributes of the concept of Spiritual Care Competence in Palliative Care, identify its antecedents and consequences, examine its empirical referents, and clarify the conceptual boundaries. The concept analysis method of Walker and Avant was used. Main attributes were organized into three main domains: (1) intrapersonal resources, such as spiritual awareness, humility, sensitivity, confidence, wisdom, and intuition; (2) interpersonal resources, such as presence, active listening, compassion, and empathy; and (3) transpersonal resources, such as the ability to establish meaningful connections, assist in finding meaning, and explore hope. These domains can be nurtured over time but ultimately require professional maturity and experience. Antecedents of spiritual care competence include active engagement in care, formal training in spiritual care, and recognition of the individual's spiritual dimension. Consequences were identified for the person being cared for, the professional and the care environment and include suffering relief, reduced stress and anxiety, enhanced spiritual well-being, and lower healthcare costs. When spiritual care competence is intentionally cultivated and continuously developed, professionals advance toward the ideal of person-centered humanistic care, fostering better at the End-of-Life patient/family outcomes and contributing to professional satisfaction and personal fulfillment.

**Supplementary Information:**

The online version contains supplementary material available at 10.1007/s10943-025-02408-1.

## Introduction

In a world undergoing technical and scientific advancement—with increasingly easy access to knowledge—healthcare has, for a long time, been dominated by the biomedical models of illness (Maslen et al., 2025). However, we are now witnessing a paradigm shift toward a more holistic approach, in which health is understood as far more than merely the absence of disease or the pursuit of a cure (Evangelista et al., 2016; Sawyer et al., 2021).

This emerging humanistic perspective embraces care as a multidimensional concept that includes spirituality as a dynamic and integrative aspect of human experience. Spirituality is inherently personal, subjective, and multifaceted. It is manifested in the search for meaning, reflection, and connection with one’s life purpose. It also involves a heightened awareness of the self and an inner relationship with the sacred—conceived as a higher power or vital force—fostering decentering and transcendence (Barbosa et al., 2016; Kitson et al., 2020). Integrating spirituality into clinical practice supports the development of a comprehensive care model that addresses the full spectrum of human needs. This model aims to enhance quality of life and overall well-being by considering the person as a whole. This shift aligns with a broader definition of “care”: one that involves moving beyond the self and focusing on the other, characterized by empathy, concern, and compassion (van Dijke et al., 2023; Zoboli, 2004).

Kierkegaard posits that people are conceived as “spirits,” asserting that genuine human life embodies a spiritual form of being (Carpintero, 2024). Although spirituality is an inherent aspect of human experience, it can be expressed and interpreted in diverse ways, depending on each person's sensitivity (Hayden et al., 2022; Mills, 2024). Providing spiritual care in today’s world is therefore a challenge, particularly within societies characterized by ideological, cultural, and religious plurality. While our shared humanity offers common ground, significant divisions remain, shaped by differing secular, spiritual, and religious orientations (Nissen et al., 2021). The Modified Biopsychosocial-Spiritual Model depicts spirituality as the core, while the outer layer includes the physical, psychological, and social levels: together they embrace the whole person (Shi et al., 2023). In this vein, Meeprasertsagool et al. (2025) propose the SPIRIT Model to highlight the core elements for spiritual care provision: “(1) Spirituality Training Programs, (2) Providers for Spiritual Care, (3) Integrating Spiritual Care into Healthcare, (4) Research and Evidence-based Practices, (5) Interdisciplinary Collaboration, and (6) Transforming Care Systems.” Research also highlights the need for greater knowledge and competence in delivering spiritual care within this interfaith context (Schipani, 2024). Spiritual care often takes place in settings where professionals responsible for care and the person being cared come from different spiritual or religious backgrounds, making it essential to develop inclusive, respectful, and responsive approaches (Liefbroer et al., 2017).

The “spiritual, religious, and existential aspects of care” are one of the eight domains outlined in the current Clinical Practice Guidelines for Quality Palliative Care by the National Coalition for Hospice and Palliative Care (NCHPC) (Clinical Practice Guidelines for Quality Palliative Care, 2018). Most End-of-Life (EoL) patients expressed that acknowledging their spiritual concerns is a crucial factor influencing healthcare decision-making, outcomes, and quality of life (Balboni et al., 2022; Rego and Nunes 2019; Puchalski et al., 2009). Despite being a key element of Palliative Care (PC), spiritual care is little acknowledged by healthcare providers and systems. Unfulfilled spiritual desires are well documented in the literature. A study conducted in the USA revealed that 97% of patients had been infrequently or never inquired about their faith over the prior year (El Nawawi et al., 2012). Pearce et al. (2012) discovered that 91% of terminally ill patients have spiritual unmet needs, and 67% sought and obtained spiritual care from their healthcare professionals. Nonetheless, 28% of patients received inadequate spiritual care, adversely affecting their emotional and spiritual well-being. More recently, Michael et al. (2023) stated that only 15% of EoL patients directly sought spiritual care, and nearly 62% identified at least one spiritual concern. The most prevalent existential concerns include fear of the dying process, loss of control, regret, need for forgiveness, guilt, loss of hope, and meaning.

Notably, over time, spirituality has been increasingly recognized as an integral dimension of total care, playing a vital role in suffering relief, as widely recognized (for example, in the PC perspective) as a practice that aims to alleviate suffering, as a threat to a person’s integrity (Lourenço et al., 2021). To address spirituality in this context of care depends on acknowledging spiritual needs: love, faith, hope, virtue, and beauty (Bartel, 2004; Fitch & Bartlett, 2019). When these needs are unfulfilled, spiritual suffering arises, making it hard for the person to find meaning, hope or connection in life. Difficult times, like facing a life-limiting illness, or even death proximity, can highlight these unmet needs, exponentiating existential suffering.

When professionals assume the spiritual care approach when they are aware of the particularities of the patient’s spirituality, integrating it into their approach and therapeutic connection, as an inseparable element of care and healing (WHO, 2016). Spiritual care is focused on the support provided with the aim of alleviating spiritual anguish and suffering, and can be subdivided into existential, psychological, religious, and social or relational dimensions (Selman et al., 2018; Monareng 2012; Tavares et al., 2022). Caring, in this specific approach, requires professionals to assume personal responsibility for others, as a moral imperative that demands altruistic care, as described by Levinas’s ethics (Benaroyo, 2022; Kong, 2008). The relationship established in this context of care views the professional as a sensitive being with vulnerabilities that can be affected by others (Kong, 2008). In a close relationship between the professional and the person being cared for, providing spiritual care includes specific interventions that contribute to spiritual well-being and quality of life—for the professional’s and the person being cared.

Recent literature identified main spiritual care interventions such as dignity therapy, life-review, meaning-centered interventions, and others such as fostering hope, meditation, mindfulness-based stress reduction, and yoga (Austin et al., 2024; de Diego-Cordero et al., 2022). If, on the one hand, professionals who perform functions within the scope of care recognize the importance of spirituality as an integral element of their clinical practice, on the other hand, they seem to lack the knowledge, inherent skills, specialized training, and time to provide spiritual care adjusted to the needs and desires of the person being cared for, resulting in gaps in theory and practice (Edwards et al., 2010; Laranjeira et al., 2022). Therefore, and considering all the above, spiritual care competence is an aptitude that fits the profile and skills to be developed by those working on care provision (Best et al., 2020; Büssing et al., 2025). This competence might pave the way to grant excellent spiritual care provision, and therefore, clarifying its definition is relevant.

The paramount elements of spiritual care encompass presence, intentionality, and compassion (Miller et al., 2023). Presence is a technique that engages patients by recognizing and respecting their holistic nature, fostering an authentic interpersonal environment, and treating patients as distinct individuals. Intentionality encompasses executing an action with empathy, aspiring to achieve the utmost benefit. Compassion entails observing an individual's lived experiences, striving to comprehend their anguish, and acting with the purpose of alleviating that suffering. To respond to these demands, evidence suggests that professionals need to be engaged in continuous spiritual development through self-care practices that sustain their own spiritual growth (Costeira et al., 2024; Matos et al., 2024). Toward that goal, they need to develop a deep self-awareness of the dimensions of self (mind, body, and spirit) that allows an inner experience of spiritual connection with the self, and integration and access to a higher conscience led by deep intuition (Laranjeira et al., 2023; Sisk, 2002). They also need to recognize one’s strengths and restraints and develop a willingness to rely on others and an openness to learn, which translates to a favorable attitude regarding spiritual issues (Costeira et al., 2024; Wolfteich et al., 2019). Lastly, reflecting on their own practice can lead to care improvement and skills acquisition.

Although there is an attempt to acknowledge spiritual care as a competence, there is a dearth of concept boundaries, leading to many different ideas of what this concept means. The responsibility for providing this type of care lies not only with chaplains or spiritual assistants, but also with health care providers (Mills, 2024). Therefore, it is important to introduce the conceptual analysis of competence in spiritual care, contributing to the clarification of the concept and its integration in practice, aiming to provide excellent care. The leads to the research question: what is the available evidence regarding the attributes, antecedents, consequences, and empirical referents of spiritual care competence?

Considering a person-centered approach, the main goal of this study is to perform a comprehensive analysis of the concept of spiritual care competence in PC. The specific aims of this study are to identify the key attributes, antecedents, consequents, empirical referents of spiritual care competence, as well as to offer an operational definition of spiritual care competence based on these attributes. Gaining a comprehensive and scientific understanding of spiritual care competence, this study seeks to provide a foundation for supportive and educational interventions to foster its development.

## Methods and Materials

### Study Design

Walker and Avant's (2019) method was used to guide an analysis of this concept. The authors proposed an interactive procedure comprising eight stages, namely (1) selecting the concept; (2) determining the purpose of the analysis; (3) reviewing the literature and identifying different uses of the concept; (4) defining attributes; (5) constructing model cases; (6) identifying antecedents; (7) identifying consequences; and (8) determining empirical referents (Walker & Avant, 2019).

### Data Sources and Analysis

A comprehensive literature review was conducted in April 2025 using the Web of Science, MEDLINE/PubMed, Cochrane, and CINAHL databases. The search methodology was derived from the Medline database and later applied to additional databases. A search string employing a mix of MeSH (Medical Subject Headings) and keywords with Boolean operators and truncation (*) was applied as follows: ([Title/Abstract] “Palliative care professional*” OR [Title/Abstract] “Health professional*” OR [Title/Abstract] “Spiritual assistant*” OR [Title/Abstract] “chaplain*” OR [MeSH term] “chaplain” OR [Title/Abstract] “clergy” OR [Title/Abstract] “nurs*” OR [MeSH term] “nurse*” OR [MeSH term] “social worker*” OR [Title/Abstract] “social worker*” OR [Title/Abstract] “psychologist*” OR [Title/Abstract] “physician*” OR [MeSH term] “physician*”) AND ([Title/Abstract]"Spiritual competen*"OR [Title/Abstract] “Spiritual care” OR [Title/Abstract] “Spiritual care competen*” OR [Title/Abstract] “Spiritual care skill*” OR [Title/Abstract] “Spiritual assistance” OR [Title/Abstract] “competen* in spiritual care”) AND (“Palliative care” OR “Hospice care” OR [Title/Abstract] “palliative assistance” OR [Title/Abstract] “Terminal care” OR [Title/Abstract] “End of Life Care” OR [MeSH term] “Palliative Care” OR [MeSH term] “Hospice Care” OR [MeSH term] “Terminal care”).

Inclusion criteria comprised publications (quantitative or qualitative studies) involving PC staff addressing intervention strategies, teaching, learning, or exercising spiritual care competence in PC and secondary studies presenting definitions or explanations of spiritual care competence. Articles that were not peer-reviewed or lacked available full text, as well as theses and dissertations, were excluded. The search is restricted to articles in Portuguese, English and Spanish and no time restrictions were imposed.

Data extraction from the articles was made using a form in Excel format, developed by the authors, containing information regarding the title, author(s), year of publication, and main findings. The information was analyzed separately by two independent reviewers who validated the inclusion/exclusion of documents (C.C. and J.C.) and a third reviewer to resolve disagreements (C.L.). Data were integrated to delineate attributes, antecedents, and consequences by a qualitative thematic analysis (Smith & Firth, 2011). Identical or analogous content was amalgamated to facilitate comparison and classification of all extracted material until the characteristics of the notion were elucidated.

## Results

**Fig. 1:**
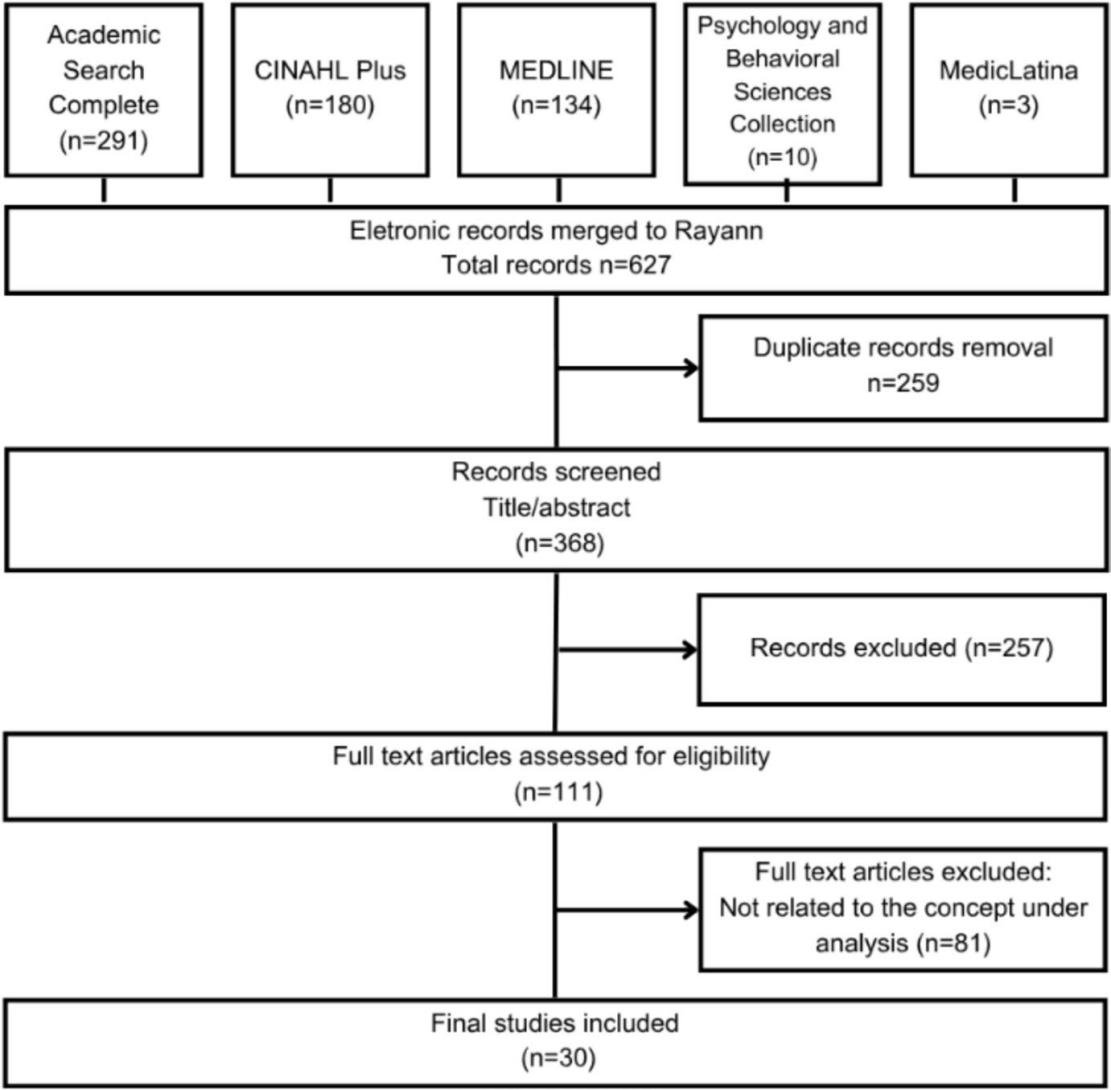
Systematic search strategy regarding concept analysis of spiritual care competence

The initial search led to the retrieval of 627 articles, 259 of which were duplicated, leaving 368 articles. Two independent reviewers screened all titles and abstracts (n = 368). After reaching an agreement on conflicts, 111 publications were selected for full-text screening, and reviewers divided the papers among themselves as previously described. In the end, 30 publications pertaining to the notion of spiritual care competency in PC were incorporated into the concept analysis (Fig. 1). The studies included in this concept analysis were published between 2008 and 2023 and originate from a range of countries: USA (n = 12), UK (n = 4), Netherlands (n = 2), Sweden (n = 1), Denmark (n = 1), Germany (n = 1), Ireland (n = 1), Iran (n = 1), Pakistan (n = 1), South Korea (n = 1), Brazil (n = 1), Australia (n = 1), and international collaborations (n = 3). In terms of type of study/article, the sample comprises ten qualitative studies, seven review articles, six mixed methods studies, three conceptual articles, three discussion papers and one white paper. The data extracted are summarized and presented in a supplementary file (Table 1)—Characteristics of the studies included in the review (n = 30).

### Use of the Concept

Spiritual care is defined as an ethical standard of professional practice that involves communicating with patients and their families, as well as developing activities and methods aimed at enhancing spiritual well-being, spiritual health, and overall quality of spiritual life (Abbasi et al., 2022). It encompasses a broad spectrum of human experience, including both religious and non-religious dimensions and is increasingly recognized as an important component of PC, built on a nonclerical model (Daaleman et al., 2008; Pagis et al., 2017). According to the *Merriam-Webster *Dictionary (2025), competence is defined as “the quality or state of having sufficient knowledge, judgment, skill, or strength to perform a specific duty or function effectively.” Thus, it can be understood as a personal attribute that contributes to superior performance. Competence integrates various dimensions that facilitate the mobilization and application of knowledge and resources, typically developed through three key domains: personal characteristics, formal training, and practical experience (Primi et al., 2001; Rodrigues et al., 2021). Moreover, competence is considered a social construct, capable of evolving over time as part of a broader theoretical framework (Fleury & Fleury, 2001).

While competence encompasses an individual’s behavioral traits and overall performance characteristics, competency refers more specifically to the demonstration of skills and abilities—where skills are understood as the capacity to perform tasks effectively, and abilities as the potential or power to act, inherent to the individual (Rodrigues et al., 2021; Salman et al., 2020). Competence, defined as the quality of being competent or properly qualified, includes a combination of key skills, knowledge, and specific attitudes (Hager & Gonczi, 1996). It reflects the relationship between one’s abilities and the successful execution of relevant tasks. Accordingly, spiritual care competence can be understood as the proper qualification and effective performance in the delivery of spiritual care, encompassing the integration of knowledge, skills, and attitudes essential for comprehensive and valuable care to address a person’s spiritual needs (Costeira et al., 2024).

### Defining Attributes

**Fig. 2:**
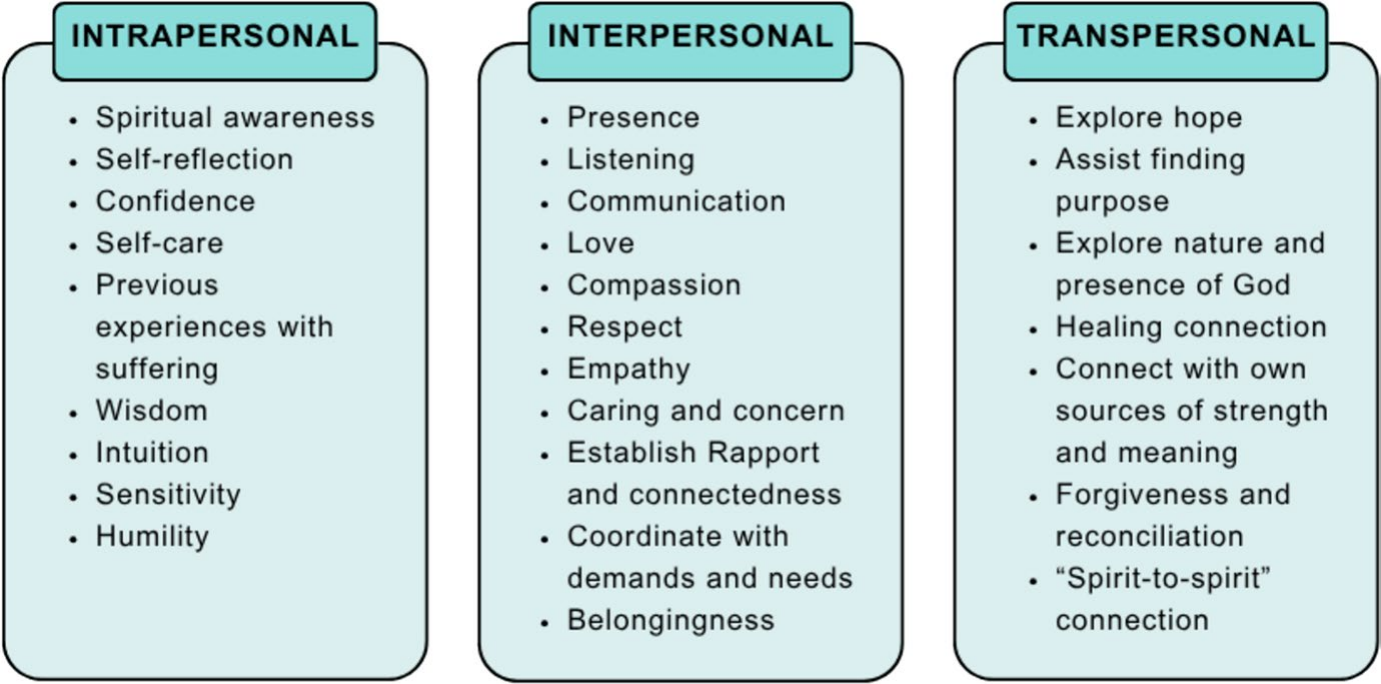
Spiritual care competence attributes

To clarify the concept under analysis, it is essential to begin by identifying its core attributes, which involves describing the specific characteristics that define the theoretical construct (Walker & Avant, 2019). This study enabled the authors to identify key attributes of spiritual care competence. These were organized into three main domains: intrapersonal resources**,** interpersonal resources, and transpersonal resources**.** These domains and their associated attributes are summarized in Fig. 2.

#### Intrapersonal Resources

Spiritual care competent professionals are spiritually aware, recognizing themselves and others as spiritual beings. They understand the importance of cultivating their own spirituality through self-reflection—enhancing their self-awareness and the therapeutic use of self—before addressing the spiritual needs of others. Previous experiences with illness and death often serve as facilitators in providing spiritual care (Baldacchino et al., 2015; Best et al., 2020; Chahrour et al., 2021; Daaleman et al., 2008; Lalani et al., 2021; Tanzi et al., 2024). To develop this specific form of awareness, mindful that personal spirituality is a strong predictor of effective spiritual care, health professionals’ benefit from engaging in self-care practices such as mindfulness, prayer, or meditative walking (Anandarajah et al., 2016; Azarsa et al., 2015; Chan, 2010).

Mindfulness, as a practice that leads to an ethically minded awareness, is a neutral self-regulatory tool. Prayer functions as a meaning-centered practice, offering consolation, hope, and existential resilience, facilitating emotional catharsis and supporting spiritual coping in painful situations. Meditative walking helps release bodily tension and clear the mind. Alongside self-care, spiritual care competence requires humility to acknowledge the subjectivity of others, while applying intuition and wisdom in the care process (Crize et al., 2018; Reed, 2014; Viftrup et al., 2021). It also includes confidence and sensitivity, essential qualities for effectively addressing spiritual needs.

#### Interpersonal Resources

To establish rapport and connectedness, competent professionals in spiritual care demonstrate strong communication skills that enable them not only to engage in meaningful conversations but also to listen attentively and nonjudgmentally (Balboni et al., 2017; Batstone et al., 2020; Bradford, 2023; Callahan, 2015; Crize et al., 2018; Daaleman et al., 2008; Ferrell et al., 2020; Lalani et al., 2021; Massey et al., 2015; Vivat et al., 2023; Watts, 2008). They also embody empathy and compassion, expressed as an active presence and a committed effort to alleviate the other’s suffering and demonstrate a genuine will to “be there” in shared encounters, showing love as a healing facilitator and concern for the person receiving care. These professionals respect the person’s views and choices and develop expertise in sensing and interpreting nonverbal bodily expressions. Even when patients are unable to verbalize their needs, they strive to establish connectedness, coordinating care to meet those needs while collaborating effectively with the care team (Abbasi et al., 2022; Best et al., 2020; Bradford, 2023; Crize et al., 2018; Lalani et al., 2021; Kang et al. 2023; Massey et al., 2015; Viftrup et al., 2021).

#### Transpersonal Resources

Spiritual competent care translates to a spirit-to-spirit connection, as a bidirectional process, where the uniquely human dimension is recognized (Reed, 2014). This connection deepens with engagement and is a form of human connection that enhances meaning. Connecting in such manner requires not only engagement and understanding of the person being cared, but also connection to one’s own sources of strength and hope (Bradford, 2023). The use of healing connection (Vivat et al., 2023), which implies holistic listening and empathic and person-centered verbal responses, is part of the process of providing competent spiritual care and might open the way for the patient to experience and integrate meaning and purpose in life (Massey et al., 2015). The availability to assist in exploring the nature and presence of God (Massey et al., 2015) is an important attribute of spiritual care competence, together with forgiveness and reconciliation. Taking part in these processes involves assisting the individual not only in the relationship with their own self but also in their path regarding the transpersonal dimension of existence.

### Case Examples

To clarify the defining criteria of the concept under analysis, a model case, a contrary case, and a borderline case will be presented, each drawn from real-life situations. To protect anonymity, pseudonyms have been used, and certain details have been modified.

#### Model Case

The model case serves as an ideal example of the concept in practice, illustrating all its defining attributes (Walker & Avant, 2019).

Paul is a highly trained PC professional with 18 years of experience in care provision. Throughout his career, he has navigated through personal losses while also supporting others through grief and suffering in his professional role. As a spiritually aware individual, Paul regularly engages in contemplative practices, fostering self-reflection, and intuition. At the end of each week, Paul gathers his team, encouraging members to share their feelings and thoughts about the past few days. These reflective meetings serve not only to build team cohesion but also to highlight the importance of addressing patients'unmet spiritual needs. On one such occasion, he shared about Anne, a 42-year-old woman and mother of an 8-year-old son, recently diagnosed with metastatic colon cancer. During the discussion, one team member remarked that Anne’s main concern was pain, and since her medications had just been adjusted, he believed her care was complete. Paul, always sensitive to team dynamics and perspectives, brought another layer of complexity to the conversation, humbly, and respectfully. He shared that although she did indeed express concerns about pain, she also conveyed feelings of loss of meaning and hope and spiritual distress. For the past two weeks, Paul had intentionally spent daily time sitting beside Anne. Using attentive listening, open communication, love, and grounded presence, he was able to establish rapport and connect with her. These interactions were marked by caring and concern, allowing Anne to feel truly seen and heard. As trust grew, through compassionate and empathic presence, she began to open up, revealing deeper spiritual needs, including a desire for forgiveness, reconciliation, and a search for inner peace. Recognizing her need to connect with her own sources of strength and meaning, Paul gently supported her in reflecting on her life, her relationships, and her sense of purpose. This “spirit-to-spirit” connection not only brought comfort to Anne but also renewed Paul’s sense of purpose. Anne soon began writing compassionate letters to her son—one for each year until his 18th birthday –wanting to leave behind something meaningful as a legacy. That same morning, she called her sister after months of silence, expressing a desire to reconcile. As Paul concluded, he invited the team to consider what more could be done to meet this woman’s spiritual needs. One team member admitted he didn’t feel equipped to address such needs, citing limited training—others agreed. In response, Paul proposed that the team complete the Spiritual Care Competence Questionnaire to identify gaps. The results revealed that spiritual self-awareness and communication competencies were among the least developed areas. Paul recommended targeted training to enhance these domains: training that would strengthen belongingness, improve their ability to coordinate with patients’ needs, and enable them to explore themes of legacy, hope, purpose, and spiritual competence. The team responded positively, appreciating the opportunity for shared learning and reinforcing the need to be involved more often in self-care and spiritual care practices.

#### Borderline Case

The borderline case includes many of the defining attributes of the concept under analysis, but not all, thereby highlighting some inconsistencies in the application of the concept (Walker & Avant, 2019).

Jane is a six-year experienced PC professional and has completed basic training in spiritual care. One day, she was called to the emergency room to support Diane, a 30-year-old woman recently referred for urgent surgery due to severe cardiac complications. Sensing her distress, Jane sat beside her, offering her full presence. She listened attentively as Diane cried, expressing how overwhelmed and terrified she felt at the thought of dying during the procedure. In fact, the surgery did not go as planned, and she was admitted to the acute care unit. When Jane visited, Diane hesitated to open up, saying, “I don’t want to bother you with my thoughts.” These words prompted Jane to gently reassure her: “I’m here for whatever you need.” This compassionate and loving gesture created a safe space for Diane to reveal her deepest fears and wishes. She spoke of her intense anxiety about death, repeatedly asking, “Why me? What have I done to deserve this?” Jane continued to listen, even though she silently struggled with not knowing how to respond or how to offer comfort. Honoring Diane’s expressed wishes, Jane coordinated with the care team to make special arrangements, allowing the husband to stay by her side without time restrictions. Afterward, she stepped outside briefly to gather herself. The emotional intensity of the situation, combined with how closely she identified with Diane—who was nearly her own age—left her feeling deeply unsettled. Recognizing the situation’s complexity but not feeling equipped to manage it, led Jane to request help, asking for a team meeting. She shared the case, hoping to reflect together on what had been done and to explore additional ways to support Diane.

#### Contrary Case

The contrary case provides a clear illustration of what spiritual care competence is not, thereby enhancing understanding of the concept by contrast and reinforcing its defining attributes (Walker & Avant, 2019).

Andrew is a physician with basic skills in PC who was responsible for Beatrice, a 79-year-old widow woman with advanced chronic obstructive pulmonary disease, living in a nursing home. Deeply religious, Beatrice often turned to her faith for comfort, even as her condition worsened. During a routine visit to manage Beatrice’s physical symptoms, Andrew was surprised when, through tears, she asked “what have I done to deserve this fate? why has God forgotten me? why is He punishing me?”. Uncomfortable and uncertain about how to respond, he quickly deflected the conversation and changed the subject. Over time, Beatrice repeatedly expressed not only physical pain but an intensifying sense of despair. Each time, Andrew dismissed her concerns, often responding curtly that she needed to wait for the medication to take effect. One day, she asked him directly, “Am I going to die?” to which he answered quite harshly: “You need to focus on today. Don’t worry so much about what’s ahead.” Desperate, Beatrice grabbed his uniform and pleaded for a visit from the priest, knowing he often came to the nursing home. She said she wanted to confess and receive blessings. Trying to leave the room, he asked her to let go. Despite Beatrice’s repeated requests over the following three days, her plea was never answered. She died alone with her spiritual needs unheard and her last wishes unmet.

### Antecedents and Consequences of the Concept

After identifying the attributes of the concept under analysis, it is important to define its antecedents, those conditions or predictive factors necessary for the development of the concept (Walker & Avant-, 2019). Prior to the emergence of spiritual care competence, professionals must be actively engaged in patient care and have received training in spiritual care (Van De Geer et al., 2016). The integration of spiritual care into healthcare services (Balboni et al., 2017; Van De Geer et al., 2016), along with the recognition of the patient's existential dimensions—including the spiritual dimension—is also essential (Abbasi et al., 2022). Additionally, the presence of spiritual sensitivity (the personal ability to pay attention to the available spiritual values in a conflicting situation and awareness of one's roles and responsibilities in that situation) (Ramezani et al., 2014) and spiritual health (possessing coping strategies to improve mental health, aid emotional control, and foster inner tranquility and comfort) are fundamental requisites for the development of spiritual care competence.

Consequences are those events that can occur as a result of the occurrence of a concept, and they are beneficial for exploring new research avenues and uncovering insights on overlooked concepts (Walker & Avant, 2019). Understanding these consequences, PC professionals can be encouraged to further engage in cultivating spiritual care skills and professional competence. These outcomes often motivate professionals to strengthen the very antecedents of the concept, such as enhancing spiritual sensitivity, improving their own spiritual health, seeking additional spiritual care training, and becoming more attentive to the spiritual needs of those in their care. This illustrates the dynamic nature of the concept, in which each action reinforces and influences the others, contributing to a continuous process of professional growth and expertise development.

Assessing and providing competent spiritual care yields significant benefits for the person receiving care, the professionals, and the overall care environment. It enables professionals to fulfill their responsibility of caring for the whole person (Watts, 2008), improving the relationship between the professional and the person being cared (Best et al., 2023), contributing to relieve suffering and support better symptom management, including reduced stress and anxiety (Crize et al., 2018). These outcomes are particularly meaningful for the person being cared, as they promote coping, resilience, and the ability to find meaning in the experience of illness. Furthermore, participation in a healing process fosters spiritual growth, contributing to both spiritual health and spiritual well-being, thereby benefiting both the professional and the person being cared. Evidence also suggests that spiritual well-being is associated with improved social, mental, and emotional health (Best et al., 2023), helps reduce burnout among professionals—especially those involved in PC—and contributes to a more positive care environment (Baldacchino et al., 2015; Batstone et al., 2020). Additionally, the assessment and provision of spiritual care have been linked to lower healthcare costs especially among minority groups and patients with significant religious coping mechanisms (Balboni et al., 2011).

### Define Empirical Referents of the Concept

The identification of empirical referents serves to demonstrate both the existence and the practical significance of the concept under analysis. There are checklists and questionnaires created for measuring spiritual care competence, such as the Spiritual Care Competence Questionnaire (SCCQ) (Frick et al., 2019) and the Spiritual Care Competence Scale (Van Leeuwen et al., 2009; McSherry et al., 2002).

SCCQ is used to assess the competences of care teams and to evaluate the outcomes of spiritual care competence training courses (Frick et al., 2019). This questionnaire has 26 items that address the perception of spiritual needs (five items), team spirit (five items), documentation competences (three items), spiritual self-awareness competences (five items), knowledge about other religions (two items), conversation competences (two items), and empowerment competences (four items). Each item is scored with a 5-point Likert scale from strongly disagree (0) to totally agree (4) (Frick et al., 2019). The SCCQ uses additional items as explanatory variables, such as barriers to spiritual care. It is currently translated and validated in ten languages.

The Spiritual Care Competence Scale is a self-assessment tool for spiritual care competence, designed in 2009 for use by nurses in clinical service areas (Van Leeuwen et al., 2009). The original scale was developed in English and is available in eight additional languages. This tool comprises six domains: assessment and implementation of spiritual care, professionalization and improving the quality of spiritual care, personal support and patient counselling, referral to professionals, and attitude toward the patient’s spirituality and communication. There are 27 questions, each scored with a 5-point Likert scale that goes from strongly disagree (1) to fully agree (5) (Machul et al., 2022; van Leeuwen & Schep-Akkerman, 2024; Van Leeuwen et al., 2009).

The instruments described above indicate not only the existence of the spiritual care competence concept but also how it can be measured. These empirical referents, set with the antecedents, attributes and consequents of spiritual care competence concept, present a clarification of the construct.

### Operative Definition

**Fig. 3:**
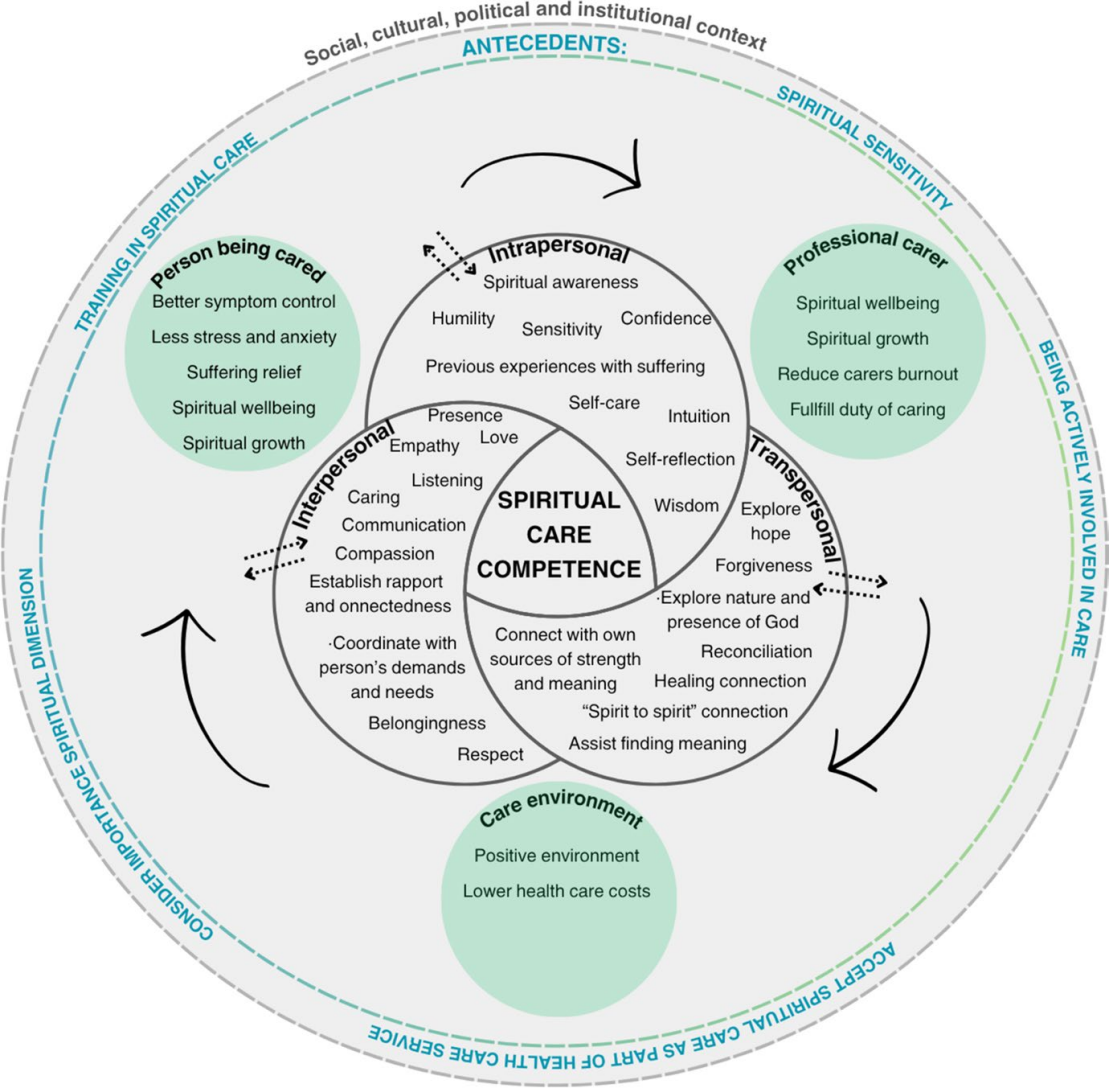
Representation of concept “Spiritual care Competence in Palliative Care”

The operative definition of the concept involves an interactive and reciprocal influence, where antecedents, attributes, and consequences continuously feed into and shape each other, creating an evolving process (Walker & Avant, 2019). Spiritual Care Competence in PC refers to a continuous and dynamic process of assessment and provision of expert interfaith spiritual care in collaboration with the multidisciplinary healthcare team. Being competent in spiritual care involves knowledge, practical skills, ethical attitudes and reflection on experience when caring for ill people and their loved ones. This competence is cultivated through spiritual sensitivity, training and intersubjective connection through a loving, empathic and compassionate posture. This connection nurtures a healing connection that translates into spiritual growth and well-being as a mutual, bidirectional outcome. This concept also recognizes that different PC professionals operate at different spiritual care competency levels. Figure 3 depicts the theoretical connection between attributes that explain spiritual care competence in PC, its antecedents and the resulting consequences.

## Discussion

This concept analysis aimed to propose a definition of spiritual care competence, a construct that emerges as a complex and interactive process. A clear understanding and definition of the concept is essential to achieve excellence in care assessment and provision. Competent spiritual care must be delivered by spiritually aware professionals who approach individuals with openness to their diverse spiritual experiences, and who are willing to establish a transcendent bond—fulfilling the moral duty of caring (Kong, 2008; Liefbroer et al., 2017).

Professionals must recognize spiritual care as a structured and intentional process. This includes the identification of spiritual needs and resources, understanding the patient's specific context, developing an individualized spiritual care plan, involving appropriate healthcare and spiritual care professionals, delivering the care, and evaluating its outcomes (Nissen et al., 2021). This process is anchored in the recognition of each person’s ontological foundation and interpreted through the “meaning-making matrix,” which reinforces the need for professionals to see the person being cared as a narrative being, requiring personalized and relational care, continuously assessed and adapted (Nissen et al., 2021).

Providing competent spiritual care is a lifelong, evolving process. The required knowledge, skills, and attitudes are acquired through formal education, clinical practice, and ongoing reflection. Equally important is the development of self-awareness, empathetic understanding of the patient's perspective, and the ability to apply tailored interventions (Baldacchino et al., 2015; Tavares et al., 2022). This expertise is not merely technical, it reflects a state of *being in doing*, achieved through the cultivation of intrapersonal, interpersonal, and transpersonal resources, expressed as knowledge, behavior, and attitudes. These domains can be nurtured over time but ultimately require professional maturity and experience (Benito et al., 2016). Gaining experience in spiritual care involves actively addressing patients’ spiritual needs and engaging authentically through therapeutic use of self. Personal beliefs, values, and levels of spiritual sensitivity shape how professionals deliver spiritual care, with life experiences enabling a deeper understanding of others (Baldacchino, 2006). Benito et al. (2016) suggest that no one can accompany another further than they have themselves gone: “our effectiveness in helping patients spiritually depends not so much on how much we know, but on who we are and how we live.” This highlights the vital importance of healthcare professionals understanding and cultivating their own spirituality as a foundation for personal and professional development. Maurya et al. (2023) propose a model of spiritual awakening that includes an initial shift in perception, sustained spiritual practice, surrender, and a continuous journey of growth. This process is rooted in vulnerability, transcendence, connection, and spiritual maturity.

Spiritual care competence enables professionals to implement targeted interventions that address patients'spiritual needs, ultimately enhancing quality of life, improving symptom control, and helping patients find meaning, purpose, and inner peace (Austin et al., 2024; Maurya et al., 2023). These outcomes often motivate professionals to reflect on and improve their own spirituality, highlighting the bidirectional nature of spiritual care—where both caregiver and care recipient are transformed.

The particularities of competent spiritual care make it clear that it is not an innate ability nor universally achievable without the necessary prerequisites. However, for those who meet the foundational requirements, spiritual care competence can be cultivated through continuous development. A significant barrier, however, is the widespread lack of training among healthcare professionals in this domain (Momeni et al., 2022). This underlines the need for institutional support and investment in spiritual care practice and education.

### Study Limitations

The present study has several limitations. One limitation identified during the development of this concept analysis is that, although much of the literature addresses spiritual care, few studies explore the concept specifically regarding competence in its provision. Another limitation is the type of databases used, which may have restricted the scope of this work. The accessed databases focused primarily on health and social sciences. In future research, sources from philosophical, theological or other relevant domains could be incorporated to broaden and deepen the analysis. Given the findings related to spiritual care competence, it would be beneficial to develop a comprehensive, transconfessional, and transcultural framework that defines levels of competence, from novice to expert levels. Such a framework would help professionals assess their current level and identify which domains (intrapersonal, interpersonal, or transpersonal) they need to develop to advance their expertise (Costeira et al., 2024). Future studies could deepen our understanding by exploring the correlation between competent spiritual care—aligned with these levels of expertise—and outcomes, such as increased spiritual well-being, positive coping mechanisms and professional quality of life.

Although spirituality is increasingly acknowledged as a core dimension of human experience, it must be more consistently incorporated into all aspects of care assessment and provision. Notably, the responsibility for delivering competent spiritual care does not lie solely with spiritual assistants, chaplains, or clergy (Mills, 2024). As emphasized in this study, spiritual care involves intrapersonal, interpersonal, and transpersonal dimensions that can be cultivated by any professional engaged in the care process. These professionals´ identification of spiritual needs, as a critical first step of spiritual care, may lead to the development of an individualized care plan or, when appropriate, to the referral to a more specialized or spiritually trained professional (Attard et al., 2019). Either way, the goal is to contribute to the provision of spiritual care ensuring the highest quality of care for the person.

### Implications for Practice

This concept analysis aligns with a broader initiative aimed at developing spiritual care guidelines for PC education and practice (Laranjeira et al., 2023). Beyond personal factors such as lack of self-awareness or limited spiritual training, professionals often cite feeling underprepared and incompetent in spiritual care provision (Matos et al., 2024). However, these barriers can be addressed through ongoing education and training, enabling a deeper understanding of spiritual care concepts and their application in clinical settings.

In this context, understanding the concept of spiritual care competence is paramount, as it equips healthcare providers to engage meaningfully in the continuous, evolving process of becoming competent in delivering this vital form of care. This analysis offers a foundational clarification of the concept, which may serve as a useful resource for those developing educational programs, clinical guidelines, or institutional strategies to support spiritual care in healthcare practice. These programs should include immersive practices such as case-based learning, role-playing, experiential training, group discussions, shared reflections, and metacognitive awareness (Başer et al., 2024; Jones et al., 2024; Meeprasertsagool et al., 2025). Such approaches can support future professionals in engaging more meaningfully with the process of developing spiritual care competence. To ensure consistency and effectiveness, it is crucial to establish clear, evidence-based guidelines for the delivery of competent spiritual care, which can be integrated into these educational initiatives.

For healthcare institutions to support the development of spiritual care competence among their professionals, it is essential to integrate spirituality into institutional policies, develop and implement spiritual care interventions; and create clinical guidelines, consensus documents, and spiritual screening tools (Ferrell et al., 2020; Henry & Timmins, 2016; Holyoke & Stephenson, 2017).

## Conclusions

Acknowledging the antecedents, attributes, and consequences of spiritual care competence is essential for delivering high-quality care. Recognizing that spiritual care competence encompasses intrapersonal, interpersonal, and transpersonal domains (each of which can be developed) empowers those involved in care provision to actively engage in their growth. Understanding the significance of spiritual care in achieving better health outcomes is a crucial first step toward developing expertise in this area. Furthermore, recognizing the positive outcomes of spiritual care competence—not only for the care environment and the individuals receiving care but also for the professionals themselves—can motivate caregivers to invest in this process. When spiritual care competence is cultivated and continuously nurtured, professionals move closer to the ideal of humanist care, leading to both professional satisfaction and personal fulfillment. In the future, specific and feasible spiritual programs to increase spiritual care competence should be developed, in order to improve professional and communication skills, as well as collaborative and person-centered practices in PC.

## Supplementary Information

Below is the link to the electronic supplementary material.

Supplementary file1 (DOCX 29 kb)

## Data Availability

The data are available from the corresponding author upon a reasonable request.

## Declarations

### Guidelines and standard

This manuscript was drafted against the eight-step model proposed by Walker and Avant (2019).
