# Supplementary material for: Spiritual Care Competence in Palliative Care: A Concept Analysis
Source: J Relig Health. 2025 Aug 8;64(5):3740–62. doi: 10.1007/s10943-025-02408-1 (PMC12449432; doi:10.1007/s10943-025-02408-1)
Supplement: Supplementary file 1 — Supplementary file1 (DOCX 29 kb) [file 10943_2025_2408_MOESM1_ESM.docx]

**Supplementary Table 1.** Characteristics of the studies included in the review (n = 30).

| Title/Author(s) | Country | Type of study/article | Aim | Sample | Main findings |
| --- | --- | --- | --- | --- | --- |
| *Spiritual Care in Palliative Care*  (Best, Vivat & Gisberts, 2023) | International/ Europe | Review article | To explore definitions of "spirituality" and "spiritual care" in palliative care contexts and summarize factors that support or hinder the integration of spiritual care into clinical practice. | 53 published articles | Spiritual care definitions; Identifies benefits of spiritual care for patients and professionals. |
| *What Do Palliative Care Professionals Understand as Spiritual Care? Findings from an EAPC Survey.*  (Vivat, Lodwick, Merino & Young, 2023) | International/ Europe | Mixed methods study | Explore how palliative care professionals perceive and implement spiritual care in their practice. | 527 palliative care professionals | Identifies main attributes of spiritual care. Refers to healing communication as an essential tool for spiritual care, highlighting the importance of good quality attention, awareness and empathy. |
| *The Nature of Religious and Spiritual Needs in Palliative Care Patients, Carers, and Families and How They Can Be Addressed from a Specialist Spiritual Care Perspective.*  Bradfords (2023) | Australia | Discussion paper | To understand how individuals facing severe or terminal illness make spiritual adjustments, discussing the integration of spiritual care principles into the biopsychosocial-spiritual model of care. | NA | Attributes of spiritual care are identified as professional competencies. Focus on transpersonal resources. |
| *Balancing hope at the end-of-life organizational conditions for spiritual care in palliative homecare in Sweden.*  (Dellenborga, 2023) | Sweden | Qualitative study | Investigate how healthcare professionals perceive, and address norms and values related to culture, religion, and spirituality when caring for patients at the end of life. | PC professionals (homecare) | Attributes of spiritual care (intra and interpersonal).  Identifies spiritual assessment in care context as a civic competence. Refers to religious literacy abilities. |
| *Spiritual Care Guide in Hospice Palliative Care*  Kang et al (2023) | South Korea | Mixed methods study | Create a spiritual care manual that reflects the fundamental and universal aspects of human spirituality. | 15 experts from hospice and PC teams | Spiritual needs assessment, a spirituality-based care continuum, a form for spiritual care records, and recommended spiritual interventions. |
| *A Thematic Analysis of Dimensions of Spiritual Care.*  (Abbasi et al., 2022) | Iran | Review article | Identify and categorize the components of spiritual care through a thematic analysis. | 79 published articles between 2013-2021 | Spiritual care is defined as an ethical performance standard and dimensions of spiritual care and specific approaches are identified. |
| *Four aspects of spiritual care: a phenomenological action research study on practicing and improving spiritual care at two Danish hospices.*  (Viftrup et al., 2021) | Denmark | **Qualitative study** | Analyze how spiritual care is perceived, experienced, and practiced by patients and staff in hospice settings, and how it can be enhanced in practice. | 12 hospice patients 9 hospice staff members | Transpersonal resources of health professionals provide spiritual care. Personalization of spiritual care according to specific needs. |
| *I Need Presence and a Listening Ear: Perspectives of Spirituality and Spiritual Care Among Healthcare Providers in a Hospice Setting in Pakistan.*  (Lalani, Duggleby & Olsen, 2021) | Pakistan | Qualitative study | Explore how healthcare providers perceive and practice spiritual care in a hospice environment. | six healthcare professionals | Definition of spiritual care. Influence of own understanding of spirituality on perceived spiritual needs and spiritual care provision in EOL care. |
| *Learning to care for the spirit of dying patients: the impact of spiritual care training in a hospice-setting.*  (Chahrour et al., 2021) | UK | Mixed methods study | Explore the perceived barriers to providing spiritual care within a hospice setting and to evaluate the impact of a research-based educational course on spiritual care among hospice staff. | 85 members of hospice staff | Development of skills needed to provide spiritual care. |
| *An EAPC white paper on multi-disciplinary education for spiritual care in palliative care.*  (Best et al., 2020) | International/ Europe | White paper | Provide guidance on integrating spiritual care education into palliative care training across Europe. | NA | Spirituality approach by health providers and its benefits. |
| *The Urgency of Spiritual Care: COVID-19 and the Critical Need for Whole-Person Palliation*.  Ferrell et al., 2020) | USA | Discussion paper | Highlight the amplified importance of spiritual care during the COVID-19 pandemic. | NA | Spiritual care attributes and implications for healthcare institutions and professionals. |
| *Spiritual care provision to end‐of‐life patients: A systematic literature review.*  (Batstone, Bailey & Hallett, 2020) | UK | Review article | Understand how nurses provide spiritual care to terminally ill patients, aiming to establish best practices in EoL care. | 11 studies with insights into spiritual caregiving practices | Spiritual needs assessment and professional carer competencies. |
| *Spiritual care at the end of life in the primary care setting: experiences from spiritual caregivers - a mixed methods study.*  (Koper et al., 2019) | Netherlands | Mixed methods study | Provide an overview of the practices of spiritual caregivers in the primary care setting and investigate, from their own perspective, the reasons why spiritual caregivers are infrequently involved in palliative care and what is needed to improve this involvement. | 31 spiritual caregivers | Professional carer skills and attitudes. Specific actions to engage in spiritual care provision. |
| *State of the Science of Spirituality and Palliative Care Research Part II: Screening, Assessment, and Interventions*  (Balboni et al, 2017) | USA | Review article | Synthesize and evaluate literature on spiritual care screening, assessment, and interventions. | NA | Describes main interventions regarding spiritual care provision. Indicates what could improve this type of care and its provision. |
| *A 10-Year Longitudinal Study of Effects of a Multifaceted Residency Spiritual Care Curriculum: Clinical Ability, Professional Formation, End of Life, and Culture.*  (Anandarajah et al., 2016) | USA | Qualitative study | Evaluate the immediate and long-term effects of a required, longitudinal spiritual care curriculum. | 26 physicians | Ability to articulate awareness of own spiritual beliefs and approach to negotiating differences between own belief and that of patients. |
| *Espiritualidade no cuidado de enfermagem ao paciente oncológico em cuidados paliativos.*  (Crize et al., 2018) | Brazil | Review article | Analyze spiritual care provided to cancer patients in palliative care, identifying the practices adopted by nursing professionals and the barriers faced. | Eight oncology patients receiving PC care | Identifies attributes of spiritual care competence. |
| *Organization-level principles and practices to support spiritual care at the end of life: a qualitative study.*  (Holyoke & Stephenson, 2017) | USA | Qualitative study | Identify organizational practices that support high-quality spiritual care at the end of life, focusing on palliative care organizations. | **46 interviews** with bereaved family members, care providers, and administrators from 4 palliative care organizations | High quality end of life spiritual care practices. |
| *Spiritual Care in Hospice and Palliative Care.*  (Ferrel, 2017) | USA | Review article | Review key aspects of spiritual care in hospice and palliative care settings. | NA | Spiritual assessment process. |
| *State of the Science of Spirituality and Palliative Care Research Part II: Screening, Assessment, and Interventions.*  (Balboni et al., 2017) | USA | Review article | Synthesize current research on spiritual screening, assessment and interventions in palliative care. | NA | Training in spiritual care, training in compassionate presence and active listening, and training in spiritual self-reflection and self-care. |
| *The Provision of Spiritual Care in Hospices: A Study in Four Hospices in North Rhine-Westphalia.*  (Walker & Breitsamete, 2017) | Germany | Qualitative study | Explore how spiritual care is provided in hospice settings and the significance of spirituality in these environments. | 22 professionals working in hospice | Professionals delivering spiritual care need to achieve connectedness with self and others and participate in continuous spiritual care training. |
| Training Spiritual Care in Palliative Care in Teaching Hospitals in the Netherlands (SPIRIT-NL): A Multicentre Trial  (Van de Geer et al. 2016) | Netherlands | **Mixed methods study** | Evaluate impact of training program in ability of healthcare professionals to address spiritual issues. | 108 Healthcare professionals from five teaching hospitals | Describes how training can contribute to competence building regarding spiritual care. |
| *An Exploration of Specialist Palliative Care Nurses’ Experiences of Providing Care to Hospice Inpatients from Minority Ethnic Groups–Implication for Religious and Spiritual Care.*  (Henry & Timmins, 2016) | Ireland | **Qualitative study** | Understand nurses' experiences in providing care to patients from minority ethnic groups within the specialist palliative care inpatient unit. | Five hospice nurses | Importance of handling one’s own beliefs before addressing others spirituality; Providing and evaluating spiritual care and integrating spirituality into institutional policy. |
| *What do I do? Developing a taxonomy of chaplaincy activities and interventions for spiritual care in intensive care unit palliative care.*  (Massey et al., 2015) | USA | Mixed methods study | Develop a standardized taxonomy of chaplaincy activities and interventions to enhance communication and understanding among interdisciplinary palliative care teams. | 67 chaplains | Identifies professional’s requirements for providing spiritual care and specific actions. |
| *Key Concepts in Spiritual Care for Hospice Social Workers: How an Interdisciplinary Perspective Can Inform Spiritual Competence.*  (Callahan, 2015) | USA | Conceptual article | Define and discuss key concepts in spiritual care. | NA | Spiritual competence enables sensitivity to patients with different religious and/or spiritual views; provision of spiritual support. |
| *Me and My Shadow: Interprofessional Training in and Modeling of Spiritual Care in the Palliative Setting.*  (Reed, 2014) | USA | Qualitative study | Investigate the impact of interprofessional training and modeling of the provision of spiritual care in palliative settings. | Palliative care unit professionals | Identifies respect as the foundation of spiritual care. Explains the spirit (of the professional) to spirit (of the patient) connection. |
| *Integrating spirituality into patient care: an essential element of person‑centered care.*  (Puchalski, 2013) | USA | Conceptual article | Define and discuss key concepts in spiritual care. | NA | Focus on health professional skills for providing spiritual care, identifying professional boundaries. |
| *Spirituality as an essential domain of palliative care: Caring for the whole person.*  (Puchalski, 2012) | USA | Conceptual article | Explore how an interdisciplinary approach can inform and enhance spiritual competence among hospice social workers. | NA | States the importance of honoring the dignity of each person and providing care that is based in compassion. |
| *The development and audit of a spiritual care policy used across three hospices in England.*  (Walters & Fisher, 2010) | UK | Discussion paper | Develop and audit a spiritual care policy that could be used across multiple hospice settings. | Hospice professionals | Focus on faith, identity, relationships and meaning. |
| *An exploratory study of spiritual care at the end of life.*  (Daaleman, 2008) | USA | Qualitative study | Explore how clinicians and other healthcare workers perceive and deliver spiritual care to dying patients and their families. | 12 Healthcare providers | Attributes of spiritual care such as presence, intention to openness, listening and loving. Identifies own personal experiences with serious illness and death as a facilitator of spiritual caregiving. |
| *Journeying with Morrie: challenging notions of professional delivery of spiritual care at the end of life.*  (Watts, 2008) | UK | **Qualitative study** | Critique professional delivery of spiritual care at the end of life and to advocate for a more holistic, community-based approach. | One terminally ill patient | Identifies attributes of spiritual care competence such as communication, listening and compassion. |

NA: Not applicable
